# Performance and eye movement patterns of industrial design students reading sustainable design articles

**DOI:** 10.1038/s41598-024-67223-2

**Published:** 2024-07-15

**Authors:** Yongchun Mao, Yanjun Han, Puhong Li, Chengming Si, Dan Wu

**Affiliations:** 1 School of Arts and Design, Qilu University of Technology (Shandong Academy of Sciences), Jinan, 250353 China; 2 School of Distance Education, Universiti Sains Malaysia, 11800 Penang, Malaysia; 3 School of Modern Logistics, Hengyang Technician College, Hengyang, 421101 China; 4 Jining Experimental High School, Jining, 272000 China

**Keywords:** Sustainable design, Design education, Industrial design, Reading strategy, Eye-tracking, Psychology, Human behaviour

## Abstract

Sustainable design education plays a crucial role in cultivating sustainability awareness and competencies among students studying industrial design. This research investigates their sustainability levels, reading performance when engaging with articles, and fixation patterns during reading. 60 industrial design students participated in the study. We evaluated their sustainability levels using the Sustainable Consumption Measurement Scale. After reading both theoretical and case article, they completed tests assessing their recall and perspective scores. We collected eye-tracking data to analyze fixation duration and conducted lag sequential analysis on fixation transitions. Students were categorized into higher and lower sustainability groups based on their sustainability scores. Female students demonstrated higher sustainability levels, and students with design experience performed better in the higher sustainability group. While recall scores did not differ significantly, the higher sustainability group exhibited elevated perspective scores in theory article. Perspective scores were generally higher for case article compared to theory article. The higher sustainability group exhibited longer fixation durations in theory article, while the case article had longer fixation durations on images. Fixation transition patterns varied between theoretical and case article, with the former featuring transitions from images to texts, and the latter demonstrating transitions between images. This study provides valuable insights into sustainable design education for students studying industrial design.

## Introduction

### Sustainable development and sustainable design

According to the World Commission on Environment and Development, sustainable development is defined as the capacity for development that meets the needs of the present without compromising the ability of future generations to meet their own needs^1^. Although the concept of sustainability has evolved, generating ongoing insights, it has achieved global dissemination and acceptance. The United Nations has set sustainable development objectives that include poverty eradication, universal healthcare access, social equity, meeting societal needs, and maintaining planetary boundaries while safeguarding future generations^2^. Nevertheless, our current production and consumption patterns deviate from sustainability principles, leading to adverse impacts on both our survival and environmental preservation^3^. Attaining sustainable development demands collaborative engagement from diverse stakeholders^4,5^. Businesses, governmental agencies, non-governmental organizations, universities, and other entities share essential responsibilities and capacities in the pursuit of sustainability. Their cooperation is vital for addressing the challenges related to sustainable development and fostering the relationship between humans and nature^6^. Each individual can contribute to sustainable development through their daily behaviors^7^. To evaluate personal sustainability levels, researchers have designed sustainability scales like the Sustainable Consumption Measurement Scale^8^, the Behaviour Scale towards Sustainable Environmental Education^9^ and the Sustainable Food Choice Questionnaire^10^. These scales assist individuals or researchers in gauging sustainability levels and thereby facilitating improvements in sustainable behaviors.

Sustainable design encompasses the practice in which designers thoroughly consider sustainability in their design processes. It aims to align the relationship between products and the environment, accounting for the influence of design choices and product usage on sustainable development, ultimately improving the sustainability of the initial product^11^. In the case of industrial products, roughly 80% of their environmental impact is determined during the product development and design stages^12^. Industrial designers hold a central role in ensuring the sustainability of products. Their responsibilities extend beyond material selection, manufacturing processes, and energy consumption; they also involve communicating design principles and influencing user behavior. In this capacity, they impact the human–environment relationship from various angles. The awareness of sustainable development among industrial designers and their capacity to promote sustainability or elevate user sustainability levels through industrial design are critical for advancing societal sustainability. Nurturing sustainability awareness and design competencies among university students majoring in industrial design is of lasting importance. This equips students with the essential knowledge, skills, and responsibilities required for sustainable design. In their future design endeavors, these students will contemplate the environmental and human well-being consequences of their design choices, devise strategies favoring sustainable development, influence consumer behavior, and consequently contribute to global sustainability^3^. Hence, incorporating sustainable design into university education programs for industrial design majors is essential. Particular attention should be devoted to the learning process of industrial design students and methods to enhance learning outcomes^13^.

### Sustainable design education

Higher education plays a crucial role in sustainability education^14,15^. A study comparing students who received sustainability education with those who did not found significant differences in their understanding and attitudes toward sustainability^16^. This demonstrates the effectiveness of such education in enhancing knowledge, attitudes, and behaviors related to sustainability. For industrial design students, given the critical role industrial designers play in product design, sustainable design education is particularly significant for global sustainable development^17^. Effective sustainable design education should be transformative, constructive, and participatory^18^. However, the current emphasis on sustainable design in design education is insufficient. Many trained designers still focus on designing to sell more products^19^. Fostering sustainable thinking and capabilities in higher education for design is crucial and urgent^20,21^. As a design concept, sustainable design education can be approached in various ways, such as reading articles on sustainable design, discussing sustainability topics^19^, and participating in sustainable design projects^22^. Reading is a common and effective learning method, widely used in university education^23,24^. In many design education activities, industrial design students read design learning materials, which is a fundamental and prevalent way to learn design. While specialized sustainable courses or projects may be effective in educating design students about sustainability, integrating sustainable concepts throughout the entire design education process is essential^18^. This highlights the importance of reading in sustainability education, as this learning method is integral to the entire design education process. Design materials that industrial design students engage with often include images and text, making the communication of design concepts and methods more intuitive. Such multimedia learning materials can also enhance students' motivation, thereby improving learning outcomes^25^. Whether for theoretical study or case studies, learning materials that combine images and text are among the most important methods for industrial design students to improve their sustainable design skills.

### Reading performance and eye-tracking studies in reading

Various methods exist for assessing post-reading performance. Some studies utilize multiple-choice questions to gauge participants' reading performance^26,27^, while others opt for the cloze test^28,29^. Furthermore, some researchers have concentrated on reading speed^30,31^. However, for some critical humanities disciplines, objective questions cannot fully reflect students' comprehension of reading materials. In a study addressing critical material reading, performance assessment entailed a post-reading test for each participant, covering two dimensions: recall and critical evaluation^24^. This approach is logical and efficacious for assessing learning outcomes in humanities disciplines where definitive answers may not exist; it can also be applied effectively for evaluation in the field of design. A study involving design and non-design students evaluated their understanding and critical thinking by using recall of perspectives (RP) and personal insights (PI) after reading critical materials^32^. For industrial design students, simply understanding the content of an article is insufficient; the ultimate goal of sustainable design education is to develop the ability to critically engage with and question the material. Therefore, the ability to understand concepts and methods related to sustainability, along with the demonstration of critical thinking during this process, are essential criteria for assessing sustainable design education.

Eye-tracking technology utilizes optical principles to record data concerning a participant's eye movements. Eye-tracking technology is widely used in reading research because it can reveal participants' visual cognition strategies, which significantly impact learning outcomes^33–35^.

Eye movements are commonly classified into sequential fixations and saccades. Fixation denotes the pause of our eyes at a specific location, whereas saccade signifies the rapid eye movement between two fixations^36^. Eye-tracking technology has been widely utilized in reading research, proficiently evaluating participants' cognitive processes and visual attention patterns throughout reading^24,27,37,38^. Among these eye movements, fixation is the most prominently discussed in eye-tracking studies related to reading. Pertinent metrics encompass fixation count and fixation duration, elucidating the extent of readers' attention to specific areas or the challenges in recognizing information within those regions^34,35,39^. To enable comparisons across distinct areas of interest, researchers categorize the materials viewed by participants into designated "areas of interest" (AOIs) guided by material attributes and experimental design. AOI analysis is a method employed to scrutinize eye-tracking data concerning diverse regions of interest^40^. By comparing the fixation count and fixation duration of different AOIs, it can be inferred which areas the participants paid more attention to during reading. This way of allocating attention is a reflection of the participants' learning style during the reading process and may affect their learning performance. Additionally, certain studies integrate lag sequential analysis^41^ into eye-tracking data, allowing the examination of gaze point shift patterns during reading and facilitating discussions regarding readers' reading strategies^24,35^. In a study on design education, lag sequential analysis was employed to examine students' visual patterns while reading multimedia design learning materials^32^. The findings indicated a correlation between visual transition patterns and learning performance. Combining the distribution of visual attention and the movement pattern of fixations, we can have a deeper understanding of the way participants read articles in reading from the perspective of cognitive psychology. Different visual modes reveal readers' reading habits, which have an impact on the effect of receiving knowledge.

### The present study

With the increasing focus on sustainable development, some scholars have recognized the importance of sustainable design education and have begun to conduct research and teaching practices targeting design students. Reading, as one of the most common learning methods, has garnered considerable attention in the educational field. Using eye-tracking data to analyze reading strategies has been validated as an effective approach. Although some studies have employed eye-tracking technology in the context of sustainable design practice^42,43^, it has not yet been utilized in sustainable design education. This is unfortunate, as eye-tracking technology has enabled significant breakthroughs in research on education and sustainable development. This study aims to fill this research gap by providing insights into visual cognition and offering pedagogical recommendations for sustainable design education.

Our study centers on undergraduate industrial design students, the prospective generation of industrial designers entrusted with product design aligned with human and environmental sustainability principles. Reading sustainable design articles constitutes a fundamental element of their sustainable design education and constitutes a key aspect of this study. We aim to ascertain whether disparities exist in industrial design students' perspectives on sustainability and their individual sustainability levels, and if so, explore the link between these disparities and sustainable design. Additionally, we endeavor to explore their eye-movement patterns during the consumption of sustainable learning materials, establishing connections between their gaze patterns, sustainability levels, and the characteristics of the learning materials.

Specifically, our study addresses three research questions (RQs):

#### RQ 1

What is the relationship between gender, prior sustainable design experience, and the sustainability levels of industrial design students?

#### RQ 2

 How do the sustainability levels of industrial design students relate to their performance after reading different types of sustainable design articles?

#### RQ 3

What are the eye movement patterns of industrial design students with varying sustainability levels when reading different types of sustainable design articles?

## Methods

### Participants

In this study, a total of 72 participants were recruited. Following the exclusion of individuals with incomplete data, the final cohort consisted of 60 undergraduate students pursuing industrial design (mean age 20.3 ± 2.45), encompassing 31 females. All participants were enrolled in a Chinese university. They had either normal or corrected-to-normal vision. Participation was voluntary, and as a token of appreciation, participants received snacks upon completing the experiment. The study was conducted according to the principles expressed in the Declaration of Helsinki and was approved by the Institutional Review Board of Qilu University of Technology. Informed consent forms were signed by all the participants before the experiment.

### Stimuli

**Figure 1:**
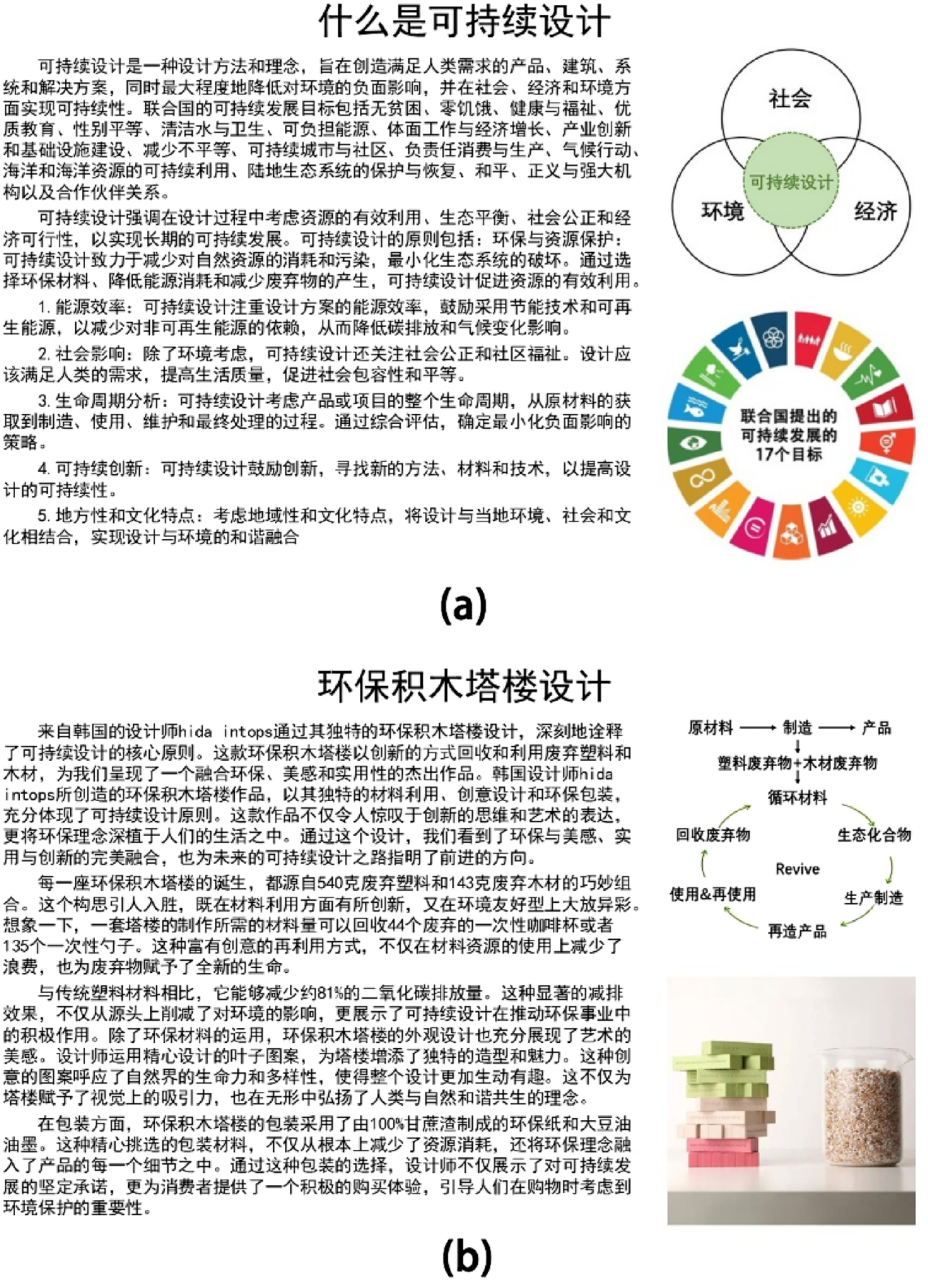
Two stimuli: (**a**) What is sustainable design? and (**b**) Eco-friendly block tower design.

**Figure 2:**
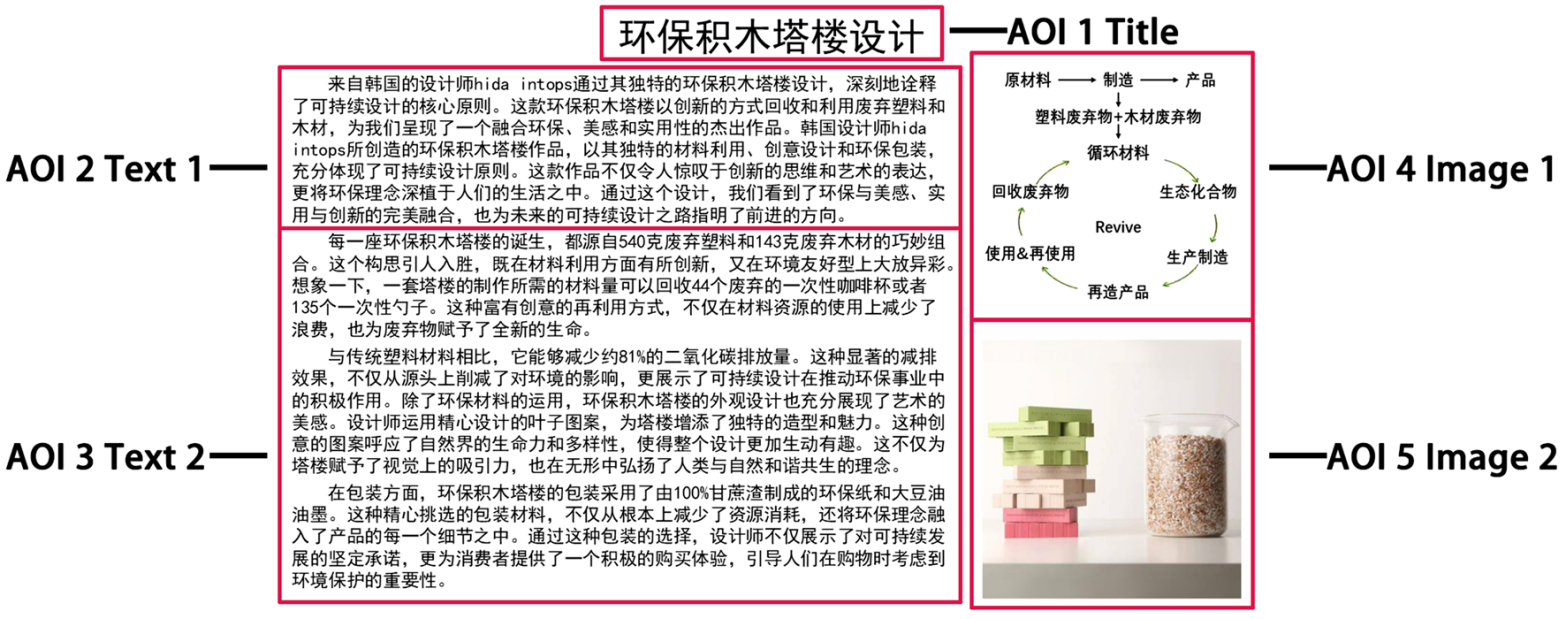
Division of AOIs in stimulus materials.

The experimental stimuli comprised two electronic articles. These articles varied in type. Stimulus 1 belonged to the theoretical category, elucidating the concept of sustainable design (Fig. 1a), while Stimulus 2 was classified as a case, introducing the design of an eco-friendly block tower (Fig. 1b). Both stimuli had the same dimensions and layouts. Each electronic article was divided into five sections (Fig. 2): AOI 1 (title), AOI 2 (text 1), AOI 3 (text 2), AOI 4 (image 1), and AOI 5 (image 2). Based on the nature of the information presented, content other than the title was classified into two groups: text and images. Text 1 served as an introduction, whereas text 2 offered detailed explanations, and the two images supplemented the textual content. The English versions of Case and Theory are in the Appendix.

### Measurements

We employed the Sustainable Consumption Measurement Scale to evaluate participants' self-assessed sustainability levels^8^. This scale encompassed 31 questions categorized into eight dimensions: activism, personal sacrifice, communitarianism, environmental concern, healthy food, perceived consumer effectiveness, search for information, and social concern. The description of the scale is provided in the Appendix. The design of this scale draws upon numerous sustainability assessment tools, offering a more comprehensive evaluation of sustainability that aligns with the latest requirements of contemporary sustainable development. For industrial designers, the eight dimensions of the scale effectively reflect the demands of sustainable development within the field of industrial design. Participants provided their responses on a 7-point Likert scale, with 1 indicating "strongly disagree" and 7 indicating "strongly agree." The scores for all questions were summed and then standardized to derive a standardized total score on a scale of 100, representing the sustainability score. The comparative fit index (CFI), Tucker-Lewis Index (TLI), and normed fit index (NFI) of this scale all exceeded 0.8, indicating a good fit between the model and observed data. Additionally, the root mean square error of approximation (RMSEA) ranged between 0.05 and 0.1, suggesting that the model reasonably accounted for potential errors.

We utilized Tobii Glasses Pro 2 to capture eye movement data. A 15-inch screen with a resolution of 1920 × 1200 displayed the two stimuli during the experiment, with the stimulus resolution set at 1280 × 794. Participants were seated in a chair while observing the stimuli and had the flexibility to move their heads and adjust their posture, but they were instructed to maintain direct facing of the screen.

Participants were required to verbally answer two questions after reading each article. Question 1: Please recall the content of the article as accurately as possible, including the issues and images. You have five minutes to give an oral report. Question 2: Based on the content of the article, present your own perspectives on sustainable design. You have five minutes to give an oral report. Sony ICD-PX470 was employed to record participants' verbal responses following stimulus observation. These recorded speeches were transcribed into text for subsequent analysis. Correct restatements of content from the article in participants' text contributed to an increment in the recall score by one point, while novel ideas pertaining to sustainable design within their text led to an increase in the perspective score. Table 1 is an example of coding recall and perspective from a participant.

**Table 1:** An example of the coding from a participant (in English).

Article	Coding		Scores	
	Recall (R)	Perspective (P)	R	P
Theory	"Sustainable design is a design concept aimed at protecting the environment and making efforts toward the sustainability of nature and society." (R#1) "Sustainable design considers the use of energy and the sustainable development of the economy in the design process." (R#2) "The United Nations has proposed 17 Sustainable Development Goals." (R#3) "Cultural considerations should be integrated into the sustainable design process." (R#4)	"Designers play a very important role in sustainable development." (P#1) "In product design, the choice of materials should not only consider aesthetics and comfort but also the potential for energy regeneration." (P#2) "In product design, emphasizing the importance of sustainability through an emotional approach is a great way to enhance users' understanding and appreciation of sustainability." (P#3)	4	3
Case	"This design uses plastic waste and wood waste as materials." (R#1) "The design achieves sustainability through the recycling of materials." (R#2) "The product packaging also uses environmentally friendly materials." (R#3) "The product design incorporates leaf patterns, which convey the idea of harmony between humans and nature." (R#4) "The use of new materials has reduced carbon dioxide emissions." (R#5)	"The colors of the building blocks can better reflect the combination of recycled materials." (P#1) "Enhancing playability in the use of the product can be considered." (P#2) "Incorporating the concept of recycling into the game might be a great idea." (P#3) "Two different versions can be launched for children and adults." (P#4) "Considering the reuse of product packaging can better practice sustainable development." (P#5)	5	5

### Procedure

**Figure 3:**
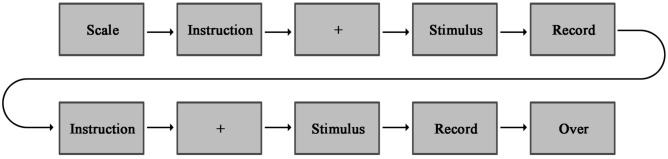
Experimental procedure.

After the researchers provided comprehensive explanations regarding the study's purpose, significance, and experimental procedures, participants gave their informed consent by signing appropriate forms. The entire experimental session lasted approximately 25 to 45 min, and you can see an illustration of the procedure in Fig. 3. Before accessing the stimulus materials, participants were required to complete the Sustainable Consumption Measurement Scale and provide demographic information, including gender, age, and whether they had prior experience in sustainable design. Following this, participants, under the guidance of the researchers, donned the eye-tracking device and, after calibration, took their seats in front of the screen. The screen displayed instructions for the eye-tracking experiment, which participants read before initiating the experiment by pressing the spacebar. After a 1000ms interval featuring a central fixation point (" + "), either stimulus 1 or stimulus 2 was automatically presented. Participants pressed the spacebar after thoroughly reading the stimulus. Subsequently, the screen displayed a new set of instructions. Participants activated the voice recorder and verbally recollected the content they had read as comprehensively as possible within a 5-min time frame. Then, they were allotted an additional 5 min to express their perspectives on sustainability. Following the recording, participants pressed the spacebar again, and the screen presented another stimulus, with participants repeating the process until the second recording was complete. To minimize potential learning effects, half of the participants commenced with stimulus 1, while the other half initiated with stimulus 2.

### Data analysis

The data collected in this study encompassed scores reflecting participants' sustainability levels, demographic information, fixation durations for each AOI, and reading performance (recall and perspective scores). Participants were classified into two groups, namely higher sustainability and lower sustainability, based on their sustainability level scores. Independent samples t-tests were employed, with gender and sustainable design experience as grouping variables, to assess disparities in sustainability levels. Furthermore, independent samples t-tests were conducted using sustainability level and type as grouping variables to explore variations in recall and perspective scores. Main effects and interaction effects were analyzed with type and content as independent variables and fixation duration as the dependent variable. A significance threshold of P < 0.05 was adopted to ascertain statistical significance. Lag sequential analysis was conducted using fixation movement trajectories, with Z > 1.96 denoting statistical significance.

## Results

The participants' average sustainability level score was 67.67, with a standard deviation of 13.877. Participants were categorized into two groups based on their sustainability level scores: the higher sustainability group (score greater than or equal to 70) and the lower sustainability group (score below 70). The higher sustainability group comprised 25 participants with an average score of 80.93 and a standard deviation of 6.946. The lower sustainability group included 35 participants with an average score of 58.20 and a standard deviation of 8.866. An independent samples t-test was conducted to compare the sustainability level scores between the two groups, revealing a significant difference (t = 10.678, p < 0.001).

The study variables encompassed categorical independent variables: gender (male and female), sustainable design experience (yes and no), sustainability level (higher and lower), type (theory and case), and content (texts and images); as well as continuous dependent variables: sustainability level, recall score, perspective score, and fixation duration (measured in seconds).

### Sustainability levels of industrial design students based on gender and sustainable design experience

To address RQ 1, we employed gender and sustainable design experience as grouping variables, and the results regarding sustainability levels are presented in Table 2. Independent samples t-tests were conducted separately for each group. The results indicated that, in the overall sample, female students exhibited significantly higher sustainability levels than their male counterparts (t = 2.208, p = 0.031). This trend was consistent in both the higher sustainability group (t = − 2.746, p = 0.012) and the lower sustainability group (t = 2.999, p = 0.005). Within the higher sustainability group, participants with sustainable design experience achieved significantly higher sustainability scores than those without such experience (t = 2.629, p = 0.015). However, in the overall sample and the lower sustainability group, no significant differences were observed (p > 0.05).

**Table 2:** Sustainability level results by gender and sustainable design experience as grouping variables.

Group	N	Sustainability score (Mean ± SD)
Higher sustainability group		
Gender		
Male	12	77.41 ± 6.164
Female	13	84.18 ± 6.151
Sustainable design experience		
Yes	12	84.33 ± 6.035
No	13	77.78 ± 6.388
Lower sustainability group		
Gender		
Male	17	54.04 ± 9.588
Female	18	62.13 ± 6.084
Sustainable design experience		
Yes	11	58.48 ± 11.343
No	24	58.08 ± 7.759
All students		
Gender		
Male	29	63.71 ± 14.305
Female	31	71.38 ± 12.584
Sustainable design experience		
Yes	23	71.97 ± 15.844
No	37	65.00 ± 11.961

### Reading performance in theory and case for higher and lower sustainability groups

To address RQ 2, we computed the results of recall score and perspective score for both the higher sustainability group and lower sustainability group (Table 3) and conducted independent-samples t-tests. The results indicate that there were no significant differences in recall scores (p > 0.05). However, significant differences were observed in perspective scores. Specifically, in the theory reading, the higher sustainability group scored significantly higher (4.28 ± 1.208) compared to the lower sustainability group (3.60 ± 1.117), t = 2.247, p = 0.028. Furthermore, in the higher sustainability group, perspective scores in the theory reading (4.28 ± 1.208) were significantly lower than those in the case reading (5.12 ± 1.453), t = − 2.223, p = 0.031. In the lower sustainability group, perspective scores in the theory reading (3.60 ± 1.117) were also significantly lower than those in the case reading (4.77 ± 1.816), t = − 3.250, p = 0.002.

**Table 3:** Recall score and perspective score for higher and lower sustainability groups.

Group	Recall score (Mean ± SD)	Perspective score (Mean ± SD)
Higher sustainability		
Theory	4.80 ± 1.443	4.28 ± 1.208
Case	4.40 ± 1.756	5.12 ± 1.453
Lower sustainability		
Theory	4.97 ± 1.339	3.60 ± 1.117
Case	4.69 ± 1.510	4.77 ± 1.816

### Fixation duration for higher and lower sustainability groups in theory and case reading

To address RQ 3, we examined the fixation duration for each AOI during reading in the higher sustainability and lower sustainability groups. The results for fixation duration in theory and case reading are presented in Table 4. During theory reading, the higher sustainability group showed significantly longer fixation duration for texts compared to the lower sustainability group (t = 5.925, p < 0.001). Additionally, the higher sustainability group exhibited significantly longer fixation duration for images than the lower sustainability group during theory reading (t = 4.037, p < 0.001). However, there were no significant differences between the two groups in fixation duration during case reading. Furthermore, we conducted an interaction effect analysis separately for the higher sustainability and lower sustainability groups.

**Table 4:** Results of fixation duration for higher and lower sustainability groups.

Group	Fixation duration (s)	
	Texts (Mean ± SD)	Images (Mean ± SD)
Higher sustainability		
Theory	169.0 ± 21.43	60.5 ± 6.88
Case	155.1 ± 20.54	113.3 ± 18.36
Lower sustainability		
Theory	137.6 ± 19.40	52.5 ± 8.01
Case	145.67 ± 21.03	105.5 ± 15.75

**Figure 4:**
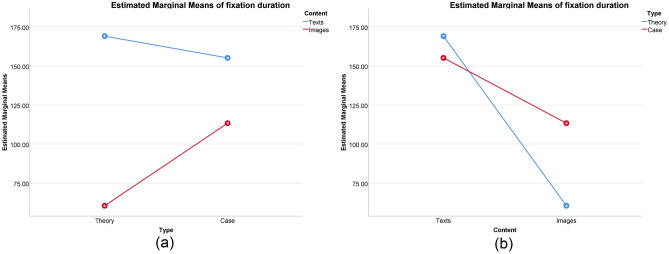
Interaction effects in the higher sustainability group: (**a**) Type as independent variable and (**b**) Content as independent variable.

In the higher sustainability group, a significant interaction effect was observed (Fig. 4). Specifically, significant main effects were found for type [F(1, 96) = 29.915, p < 0.001, η^2^ = 0.238] and content [F(1, 96) = 446.534, p < 0.001, η^2^ = 0.823], along with a noteworthy interaction effect between type and content [F(1, 96) = 87.972, p < 0.001, η^2^ = 0.478]. Simple effects analysis revealed that fixation duration for texts in theory was significantly longer than in case [F(1, 96) = 7.643, p = 0.007, η^2^ = 0.074; mean difference = 13.908]. Fixation duration for images in theory was significantly shorter than in case [F(1, 96) = 110.244, p < 0.001, η^2^ = 0.535; mean difference = − 52.820], and fixation duration for texts in theory was significantly longer than for images in theory [F(1, 96) = 465.451, p < 0.001, η^2^ = 0.829; mean difference = 108.532]. Moreover, fixation duration for texts in case was significantly longer than for images in case [F(1, 96) = 69.055, p < 0.001, η^2^ = 0.428; mean difference = 41.804].

**Figure 5:**
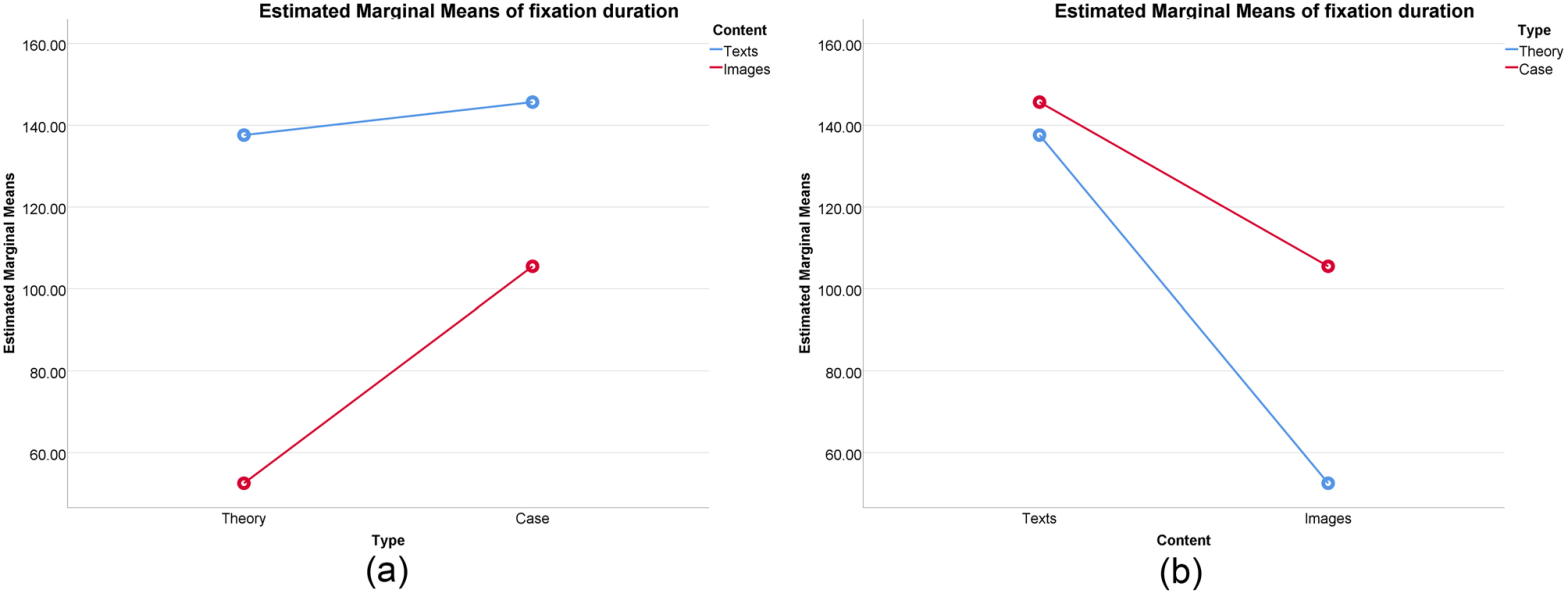
Interaction effects in the lower sustainability group: (**a**) Type as independent variable and (**b**) Content as independent variable.

In the results of the lower sustainability group, a significant interaction effect was observed (Fig. 5). Specifically, there was a significant main effect of type [F(1,136) = 115.364, p < 0.001, η^2^ = 0.459], and a significant main effect of content [F(1,136) = 485.470, p < 0.001, η^2^ = 0.781]. Additionally, the interaction between type and content was also significant [F(1,136) = 62.426, p < 0.001, η^2^ = 0.315]. The results of simple effects analysis indicated that fixation duration for texts in theory was significantly lower than that in the case [F(1,136) = 4.032, p = 0.047, η^2^ = 0.029], with a mean difference of -8.071. Fixation duration for images in theory was significantly lower than that in the case [F(1,136) = 173.758, p < 0.001, η^2^ = 0.561], with a mean difference of -52.986. Fixation duration for texts in theory was significantly higher than that for images [F(1,136) = 448.034, p < 0.001, η^2^ = 0.767], with a mean difference of 85.083. In the case, fixation duration for texts was significantly higher than that for images [F(1,136) = 99.862, p < 0.001, η^2^ = 0.423], with a mean difference of 40.169.

### Fixation transition in the higher and lower sustainability groups in theory and case reading

**Figure 6:**
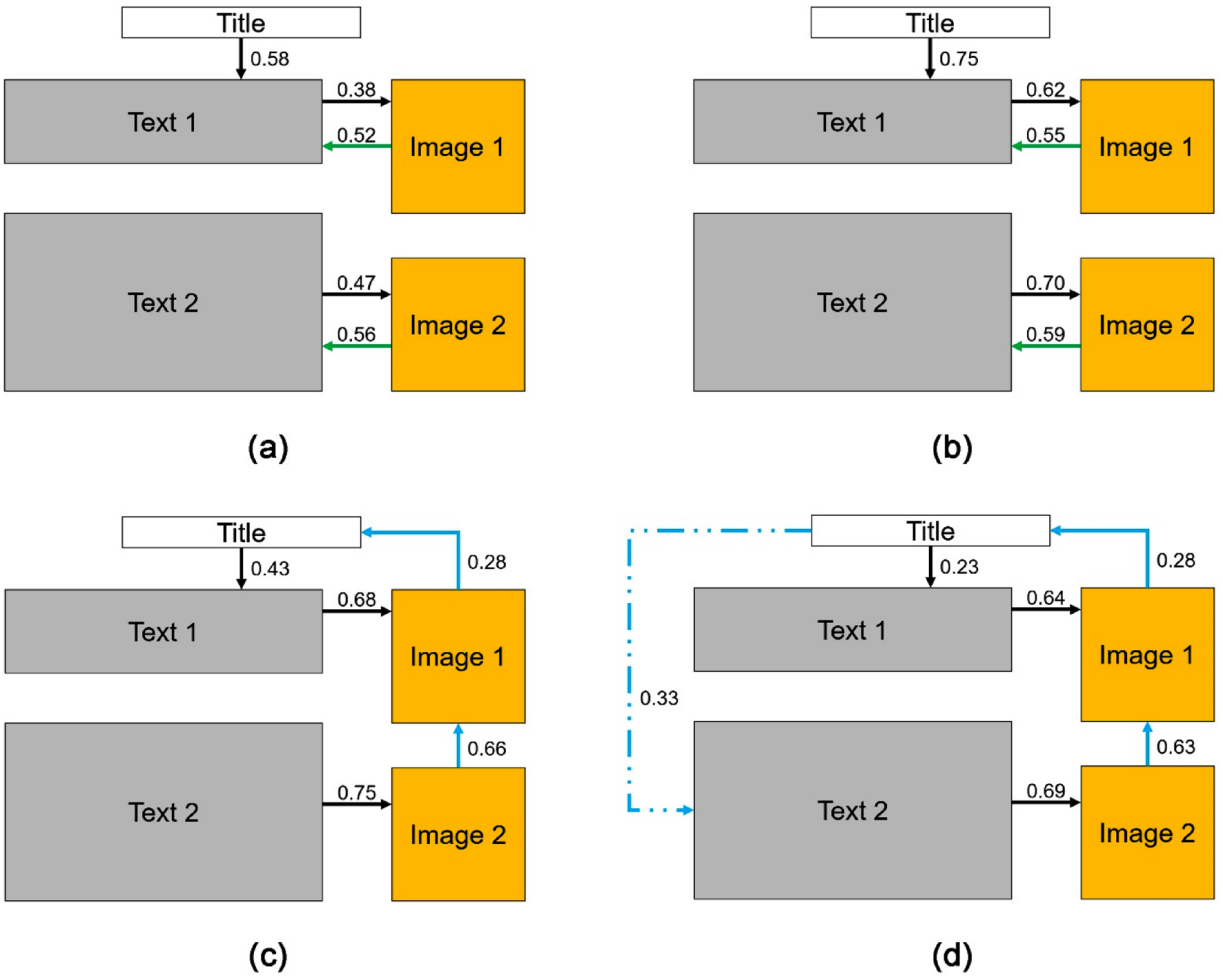
Fixation transition patterns of the two sustainability groups (higher and lower) reading the two types of articles (theory and case): (**a**) Higher—theory, (**b**) Lower- theory, (**c**) Higher s- case, and (**d**) Lower—case.

In order to further investigate RQ 3, we conducted lag sequential analyses of fixation transitions separately for the higher sustainability group and the lower sustainability group during theory and case readings. In theory reading, both groups displayed similar and significant fixation transitions (Table 5). These transitions included movements from AOI 1 to AOI 2, AOI 2 to AOI 4, AOI 3 to AOI 5, AOI 4 to AOI 2, and AOI 5 to AOI 3. During case reading, both groups exhibited similar significant fixation transitions, including movements from AOI 1 to AOI 2, AOI 2 to AOI 4, AOI 3 to AOI 5, AOI 4 to AOI 1, and AOI 5 to AOI 4. However, the lower sustainability group showed a significant transition from AOI 1 to AOI 3, which was not observed in the higher sustainability group (Table 6). For a visual representation of these differences and connections in fixation transition patterns, please refer to Fig. 6. Various colors in the figure denote differences in students' readings of different article types within the same sustainability level. Distinct arrow styles represent differences in students' readings of articles of the same type between different sustainability levels. Fixation transitions exhibited variations between different article types. In comparison to theory article, case article had additional transitions from AOI 4 to AOI 1 and AOI 5 to AOI 4 but lacked transitions from AOI 4 to AOI 2 and AOI 5 to AOI 3. Notably, the lower sustainability group displayed an additional transition from AOI 1 to AOI 3.

**Table 5:** Z-values for the fixation sequence analysis in theory reading for the higher and lower sustainability groups (* means Z > 1.96, p < 0.05).

AOI	Sustainability level	AOI 1	AOI 2	AOI 3	AOI 4	AOI 5
AOI 1	Higher	0	4.43*	− 2.88	− 0.32	− 1.78
	Lower	0	6.24*	− 2.42	− 1.69	− 3.33
AOI 2	Higher	− 0.5	0	0.5	3.62*	− 3.78
	Lower	− 0.21	0	− 2.07	10.22*	− 8.09
AOI 3	Higher	− 0.18	− 2.81	0	− 2.16	5.74*
	Lower	0.41	− 5.51	0	− 6.57	13.43*
AOI 4	Higher	− 0.16	2.95*	− 2.68	0	− 0.38
	Lower	1.35	3.24*	− 1.83	0	− 2.7
AOI 5	Higher	0.98	− 3.73	4.71*	− 1.68	0
	Lower	− 1.63	− 1.37	6.36*	− 4.44	0

**Table 6:** Z-values for the fixation sequence analysis in case reading for the higher and lower sustainability groups (* means Z > 1.96, p < 0.05).

AOI	Sustainability level	AOI 1	AOI 2	AOI 3	AOI 4	AOI 5
AOI 1	Higher	0	5.2*	0.29	− 4.82	0.97
	Lower	0	2.65*	3.62*	− 4.15	− 0.55
AOI 2	Higher	− 2.17	0	0.71	4.21*	− 3.85
	Lower	− 1.14	0	− 0.35	5.3*	− 4.52
AOI 3	Higher	− 1.88	− 1.49	0	− 4.68	7.68*
	Lower	− 0.29	− 1.17	0	− 6.84	8.37*
AOI 4	Higher	4.56*	0.36	− 0.21	0	− 3.1
	Lower	3.44*	1.59	− 0.87	0	− 3
AOI 5	Higher	− 1.83	− 2.89	− 0.54	3.52*	0
	Lower	− 2.57	− 2.78	− 1.64	5*	0

## Discussion

To address the three research questions, we conducted sustainability level assessments on 60 industrial design students, tested their recall and perspective scores after reading both theory and case article, and simultaneously recorded their eye-tracking data. For RQ 1, we observed that female students exhibited higher levels of sustainability. Additionally, students with sustainable design experience obtained higher scores in the higher sustainability group. Regarding RQ 2, we found no significant difference in recall scores. However, during theory reading, the higher sustainability group demonstrated an advantage in perspective scores. Notably, industrial design students performed better in perspective scores when reading case article compared to theory article. Turning to RQ 3, we examined our findings in terms of fixation duration and fixation transition. In terms of fixation duration results, the higher sustainability group allocated more time to both text and image areas during theory reading compared to the lower sustainability group. We also noted significant interaction effects between article type and content, which were consistently observed in both the higher and lower sustainability groups. However, there were distinctions between them. Specifically, the higher sustainability group exhibited longer fixation durations for text in theory reading compared to text in case reading, while the opposite trend was observed in the lower sustainability group's results. Moreover, the lag sequential analysis results indicated that fixation transition patterns were largely similar between the higher and lower sustainability groups. Nevertheless, differences emerged when considering different article types. We will elaborate further on these findings in the subsequent discussions.

In our study, we observed that female students exhibited higher levels of sustainability in comparison to their male counterparts. This observation aligns with previous research findings indicating that females tend to manifest higher sustainability levels, and notably, this difference appears to widen as individuals grow older^44^. A study conducted in Spain similarly reported that females tend to display heightened sensitivity to sustainability concerns^45^. The consistency between our findings and prior research suggests that the gender-based disparity in sustainability among industrial design students in China mirrors broader global trends. The differences arising from gender may stem from family, school, or the students themselves. Gender socialization begins in early childhood and is reinforced by parents and schools^46^. The role expectations for girls encourage their sensitivity to relationships with others and society, which enhances their awareness of sustainability issues^44^. Among all students, those with prior sustainable design experience did not demonstrate a significant advantage in sustainability levels, except within the higher sustainability group. Previous studies have already established that active engagement in related activities, particularly interactive projects or initiatives, can notably heighten awareness and sensitivity to sustainability issues^47,48^. However, for individuals in the lower sustainability group, we interpret these results as indicating that their involvement in sustainable design projects may not be driven by intrinsic motivation or a genuine commitment to sustainability principles. Instead, their participation might be primarily motivated by external incentives such as credits or financial rewards, which may not necessarily result in an actual improvement in their sustainability levels.

Based on these findings, educators need to consider gender differences and the timing of design practice in sustainable design education. Given these differences, it is essential to assess the educational effectiveness of sustainable design separately for male and female students to avoid overlooking the lower awareness of sustainability among boys. Moreover, while educators recognize the benefits of sustainable design practice in cultivating sustainability, the timing of such practices is equally important and often overlooked. Engaging in design practice when students' awareness and capabilities in sustainability are at a low level might not be effective, as the impact could be minimal. Given the limited time available for design education at universities, a more effective approach would be to involve students in sustainable design projects after they have received sufficient education in sustainable design and their sustainability awareness has reached a higher level.

Improving academic skills through reading theoretical or case articles is a common approach. Understanding the content of an article and being able to recall it is fundamental to mastering theories and skills, which can be measured through immediate recall after reading^24,32^. Furthermore, the ability to propose new perspectives based on the article's content reflects students' critical thinking and innovation skills, which are crucial for industrial design students. We evaluated the performance of industrial design students in comprehending sustainable design sustainable design articles, focusing on two aspects: recall score and perspective score. The recall score did not exhibit significant differences, neither between the higher sustainability group and the lower sustainability group nor between theory and case article. Although prior knowledge is acknowledged to have an impact on memory retention^49^, the sustainability levels may not necessarily correspond to the students' proficiency in sustainable design knowledge. When engaging with sustainability design articles in university, industrial design students, regardless of their inherent sustainability awareness, demonstrate a substantial ability to comprehend and recall the article's content. The type of article, whether theoretical or case-based, does not affect their understanding. It is important to note that to convert this information into long-term memory, it is necessary to review it repeatedly, use various memory strategies (such as association, organization, and meaningful linkage), and apply this information in different contexts. Concerning perspective score, in theory article, the higher sustainability group displayed an advantage, while there was no notable difference in case article. Higher sustainability students appear to be more open to sustainability-related concepts and better equipped at constructing meaning through imagination when engaging with theoretical content^50^. Furthermore, irrespective of sustainability levels, perspective scores were higher for case article when compared to theory article. Extensive research has established that, in contrast to theoretical content, more vivid and practical case studies are more effective in stimulating divergent thinking^51,52^, a benefit that is particularly pronounced among students majoring in industrial design^53,54^.

Although recall is crucial for university students' learning, the results of this study suggest that it is not the core factor in developing sustainable design skills. In the comparison between case-based and theory-based learning, case-based learning demonstrated a significant advantage in terms of learning outcomes. However, this does not imply that case-based learning can completely or partially replace theory-based learning in sustainable design education. The higher performance of the high sustainability group in theory-based learning underscores the importance of theoretical understanding, despite the greater difficulty students face in comprehension and divergent thinking. The traditional approach in design education, which predominantly emphasizes learning theory before cases, warrants reconsideration. While this method has proven effective in many disciplines, it may not be the most suitable for design education. An alternative approach that introduces foundational theories followed by case-based learning, with deeper theoretical engagement after students have developed a certain level of understanding, is worth exploring for design educators and reformers.

Fixation duration serves as an indicator of the degree of attention devoted to specific areas^35,36,38^. In theory article, the higher sustainability group exhibited a more pronounced focus on both text and images in comparison to the lower sustainability group. This heightened attention is further reflected in the higher perspective scores achieved by the higher sustainability group in theory article. In contrast, sustainability levels did not significantly impact students' reading behaviors in the case article. Notably, a significant interaction effect between article type and content was evident, consistently observed in both the higher sustainability and lower sustainability groups. These results showcased several commonalities between the two groups. Specifically, regardless of whether they were reading theory or case article, students tended to concentrate more on textual content than images. Furthermore, in the context of case article, students exhibited a heightened focus on images compared to theory article. This tendency can be attributed to the nature of textual content, which typically conveys a greater volume of information than images^55^. Consequently, processing textual information incurs a higher cognitive load^25^, Consequently, processing textual information incurs a higher cognitive load^35,56^. Students majoring in design are more sensitive to design project cases and are more interested in engaging with them^53,54^. In the case article, students exhibited a notably elevated level of attention to images. This heightened attention correlated with the superior perspective scores attained in case article. In essence, students specializing in design were more attuned to the nuances of design project cases, fostering a deeper engagement with and extraction of information from the accompanying images. Consequently, this translated into an increased time investment in observing images and the generation of novel ideas. Sustainability levels were significantly associated with fixation duration concerning textual content. Specifically, the higher sustainability group demonstrated a heightened concentration on text within theory article compared to case article. Conversely, within the lower sustainability group, attention to case article surpassed that in theory article. The higher sustainability group exhibited a more pronounced inclination to devote extended periods to textual examination within theory article, with fixation durations even surpassing those observed in case article. This phenomenon can be attributed to the fact that theory article contains a more substantial volume of information embedded within the text. However, the sustainability levels of students may exert an influence on their interest and energy allocation toward textual content in theory article, ultimately culminating in enhanced reading performance within this category for the higher sustainability group.

The fixation transition pattern offers valuable insights into the reading strategies employed by both student groups when engaging with theory and case article^24,35^. Figure 6 provides a clear visualization of the commonalities and distinctions between these groups and across article types. The progression from the title to Text 1 adheres to a top-down reading order^33^, a consistent feature across all results. Similarly, the pathways from Text 1 to Image 1 and from Text 2 to Image 2 emerge due to their inherent semantic connections^57^ and follow a left-to-right sequence^58^, thus manifesting in all outcomes. In theory article, the fixation transition pattern remains consistent between both student groups, except for the previously mentioned exceptions, notably the pathways from Image 1 to Text 1 and from Image 2 to Text 2, which are absent in case reading. This divergence is attributed to the distinctive characteristics of theory article, housing more abstract information that necessitates an interactive reading approach, requiring students to navigate between textual and visual elements. In contrast, during case reading, students' fixations shift from texts to images without returning, as they can more efficiently extract information from images without relying on textual support. Furthermore, a notable observation in case reading is the pathway from Image 2 back to Image 1, signifying that students can establish connections between images in case article and extract meaningful information^59^. The case article also exhibits a pathway from Image 1 to the title, a feature absents in the theory article. This phenomenon likely arises from the greater alignment between the case article title and accompanying images, enhancing their interrelatedness. The sole divergence between the two student groups emerges in case reading, with the lower sustainability group demonstrating a pathway from the title to Text 2, absent in the higher sustainability group. While the precise cause of this discrepancy remains uncertain, it is speculated that it results from fixations transitioning from Image 1 to the title, subsequently progressing to Text 2 for further reading.

In the study materials, images and text convey information differently, and distinct reading patterns were observed between the high-sustainability group and the low-sustainability group. These differences can offer valuable insights for design educators. While images tend to capture students' attention more easily and students appear to prefer case-based articles, deeper content is often found in the text and theory-based articles. This preference for more vivid content suggests that educators should employ various educational techniques to encourage students to engage more with textual and theoretical material. Furthermore, the way high-level students shift their visual attention between text and images differs from that of low-level students, potentially contributing to the variance in sustainability comprehension levels. Educators should consciously train students to revisit the text after viewing images, as this practice can foster deeper reflection.

Sustainable development education is a crucial component of higher education, especially in the context of the current global energy crisis. Educators should pay attention to sustainable design education for industrial design students. However, few studies analyze and discuss sustainable education for industrial design students from the perspectives of learning processes, performance, and sustainability levels. This study delved into the sustainability levels, reading performance of industrial design students when engaging with sustainable design articles, and their eye-tracking patterns, yielding some noteworthy findings. These findings can provide insights for educational reform in industrial design programs, classroom teaching, and skill development for industrial design students. We believe that the improved industrial design education can effectively enhance the overall sustainability level of industrial design students. Enhancing the sustainability awareness of industrial design students will be reflected in their future design works, undoubtedly contributing to global sustainable development. This study offers specific recommendations for design educators and introduces new perspectives for research on sustainable education. Design educators should focus on gender differences in sustainable design education, the role of sustainable design practice, and the impact of the type and content of learning materials on students' reading strategies. These areas still have unresolved and unexplored issues that require further research in future studies.

It is essential to acknowledge several limitations. Firstly, the sample was exclusively drawn from industrial design students at a single Chinese university, which might not comprehensively represent all industrial design students. In future research, the sampling scope should be expanded. Secondly, the assessments of sustainability levels and reading performance relied on subjective measures, lacking objective means of evaluation. The assessment of reading performance considered two indicators: recall and perspectives. However, these two indicators cannot fully reflect the students' outcomes. Future research will explore more comprehensive and reasonable indicators and consider adopting a longitudinal study approach. Thirdly, the sustainable design learning materials utilized in the experiment represented a specific style, rendering it challenging to extrapolate the findings to all sustainable design education contexts. Future research should also examine different learning materials, learning methods, and the process of engaging in design practice for industrial design students. Lastly, while the study unearthed certain reading behavior patterns of industrial design students when interacting with sustainable design articles, the limited prior research in this domain leaves some results not fully explicable at this time. We anticipate that future scholars will join us in further exploring this realm, ultimately advancing sustainable design education.

## Implications

This study offers valuable insights into sustainable design education within industrial design programs. The observed gender differences in sustainability levels^44^ underscore the importance of addressing disparities between male and female students in sustainable design education. Further research is needed to comprehend the reasons behind the tendency for females to exhibit higher levels of sustainability awareness. Engaging in sustainable design projects has proven beneficial for elevating sustainability levels, as active participation leaves a more profound impact^47,48^. Consequently, universities and educators should consider providing additional practical opportunities for students to enhance their sustainability awareness. Industrial design students exhibit a heightened interest in reading design case article and generate more perspectives compared to theory article. The integration of design cases into design education can be particularly advantageous^53^. Notably, higher sustainability students demonstrate more prolonged fixation durations in theory article, resulting in superior reading performance. This presents a promising avenue for future research. Diverging from traditional design education processes, an approach commencing with reading design cases and engaging in design practice to cultivate sustainability awareness before delving into more profound sustainability theory warrants exploration. Within the context of industrial design students, it becomes evident that they allocate more attention to images in case article than in theory article, engaging in interactions between these images. Consequently, their performance in reading case article excels. This underscores the significance of incorporating visual materials into learning resources, as research has shown it to be more effective for students' learning outcomes^35,60^. Hence, sustainable design education for industrial design students should prioritize this aspect.

### Supplementary Information

Supplementary Information.

## Data Availability

The data that support the findings of this study are available from the corresponding author upon reasonable request.
