# Supplementary material for: Performance and eye movement patterns of industrial design students reading sustainable design articles
Source: Sci Rep. 2024 Jul 15;14:16267. doi: 10.1038/s41598-024-67223-2 (PMC11251013; doi:10.1038/s41598-024-67223-2)
Supplement: Supplementary file 1 — Supplementary Information. [file 41598_2024_67223_MOESM1_ESM.docx]

**Appendix 1 Article (Theory) in English**

What is sustainable design?

Sustainable design is a design approach and philosophy that aims to create products, buildings, systems, and solutions that meet human needs while minimizing negative environmental impacts and achieving social, economic, and environmental sustainability. The UN's Sustainable Development Goals include No Poverty, Zero Hunger, Health and Well-being, Quality Education, Gender Equality, Clean Water and Sanitation, Affordable Energy, Decent Work and Economic Growth, Industrial Innovation and Infrastructure Development, Reduced Inequalities, Sustainable Cities and Communities, Responsible Consumption and Production, Climate Action, Sustainable Use of Oceans and Marine Resources, Conservation and Restoration of Terrestrial Ecosystems, Peace, Justice and Strong Institutions, and Partnerships.

Sustainable design emphasizes the consideration of efficient use of resources, ecological balance, social justice, and economic feasibility in the design process to achieve long-term sustainable development. The principles of sustainable design include Environmental protection and resource conservation: Sustainable design is committed to reducing the consumption and pollution of natural resources and minimizing the damage to ecosystems. Sustainable design promotes the efficient use of resources through the selection of environmentally friendly materials, reduced energy consumption and reduced waste generation.

1.Energy efficiency: Sustainable design focuses on the energy efficiency of design solutions and encourages the adoption of energy-efficient technologies and renewable energy sources to reduce dependence on non-renewable energy sources, thereby reducing carbon emissions and climate change impacts.

2. Social impact: In addition to environmental considerations, sustainable design also focuses on social justice and community well-being. Design should meet human needs, improve quality of life, and promote social inclusion and equality.

3. Life Cycle Analysis: Sustainable design considers the entire life cycle of a product or project, from the acquisition of raw materials to manufacturing, use, maintenance, and final disposal. Strategies to minimize negative impacts are identified through a comprehensive assessment.

4. Sustainable Innovation: Sustainable design encourages innovation and the search for new methods, materials, and technologies to improve the sustainability of design.

5. Local and cultural characteristics: Considering regional and cultural characteristics, the design is combined with the local environment, society, and culture, so as to achieve the harmonious integration of design and environment

**Appendix 2 Article (Case) in English**

Eco-friendly building block tower design

South Korean-based designer Hida Intops profoundly interprets the core principles of sustainable design through her unique eco-friendly building block tower design. This eco-friendly building block tower recycles and reuses waste plastic and wood in an innovative way, presenting us with an outstanding piece that blends eco-friendliness, aesthetics, and practicality. Korean designer Hida Intops has created eco-friendly building block towers that embody sustainable design principles with their unique use of materials, creative design, and eco-friendly packaging. This work is not only amazing for innovative thinking and artistic expression, but also deeply rooted the concept of environmental protection in people's lives. Through this design, we see environmental protection and beauty.

With the perfect integration of innovation, it also points out the way forward for the future sustainable design. Each eco-friendly building block tower is born from a clever combination of 540 grams of waste plastic and 143 grams of waste wood. It's a fascinating idea that is both innovative in terms of material use and environmentally friendly: imagine that a tower could recycle 44 discarded single-use coffee cups or 135 disposable spoons in the amount of material needed to make a tower. This creative way of reusing not only reduces waste in the use of material resources, but also gives new life to waste.

It is able to reduce CO2 emissions by about 81% compared to conventional plastic materials. This significant emission reduction effect not only reduces the impact on the environment at the source, but also demonstrates the positive role of sustainable design in promoting environmental protection. In addition to the use of environmentally friendly materials, the exterior design of the eco-friendly building block tower also fully demonstrates the beauty of art. The designers used an elaborate leaf pattern to add a unique shape and charm to the tower. This creative pattern echoes the vitality and diversity of the natural world, making the whole design livelier and more interesting. This not only gives the tower a visual appeal, but also invisibly promotes the concept of harmonious coexistence between humans and nature.

In terms of packaging, the packaging of the eco-friendly building block tower adopts eco-friendly paper and soybean oil ink made of 100% bagasse. This carefully selected packaging material not only radically reduces resource consumption, but also integrates environmental protection into every detail of the product. With this choice of packaging, the designer not only demonstrates a strong commitment to sustainability, but also provides consumers with a positive buying experience that leads people to consider the importance of environmental protection when shopping.

**Appendix 3 Sustainable Consumption Measurement Scale**

| NO. | Dimension | Description |
| --- | --- | --- |
| 1 | Activism | I believe people should encourage their friends not to use products that pollute or harm the environment. |
| 2 |  | I try to influence the consumption of people in my social circle, even if they do not share the same values. |
| 3 |  | I participate in activities of environmental preservation. |
| 4 |  | Whenever I see people consuming in a manner I do not consider adequate, I question them. |
| 5 | Personal sacrifice | I would be willing to increase my expenses by 20% to use natural resources more adequately. |
| 6 |  | I usually make conscious efforts to limit the use of products made from scarce resources. |
| 7 |  | I am willing to make personal sacrifices to reduce pollution, even if immediate results are not significant. |
| 8 |  | I consider the environmental potential of my actions in many of my decisions. |
| 9 | Communitarianism | I avoid doing all my shopping in large stores (big retailers and multinational companies). |
| 10 |  | I avoid consuming industrially produced items as much as possible. |
| 11 |  | I try to help local commerce in my region when I shop. |
| 12 | Environmental concern | I refrain from buying products that have too much packaging. |
| 13 |  | I substitute products I usually buy for ecological reasons. |
| 14 |  | I change my shopping habits because of my concern with the environment. |
| 15 |  | I avoid using products or services that harm the environment. |
| 16 |  | I generally buy products that come in recyclable packaging. |
| 17 |  | I generally buy products that pollute less. |
| 18 | Healthy food | I try to choose food without additives. |
| 19 |  | I try to have a healthy eating habit. |
| 20 |  | I try to eat as little processed/industrialized food as possible. |
| 21 | Perceived consumer effectiveness | I believe every consumer can have a positive effect on society by buying products from socially responsible companies. |
| 22 |  | I believe all consumers should be interested in the environmental consequences of the products they buy. |
| 23 |  | I believe that I can influence people to consume in a better way through my example. |
| 24 | Search for information | I try to attend conferences about sustainability. |
| 25 |  | I try to read newspapers, magazines, and blogs about sustainability. |
| 26 |  | I have found information online that has made me change my consumption pattern. |
| 27 | Social concern | I usually buy products from companies that help victims of natural disasters. |
| 28 |  | I usually buy products from companies that hire people with special needs. |
| 29 |  | I generally buy products from companies that help underprivileged people. |
| 30 |  | I usually buy products whose profits are partially donated to humanitarian causes. |
| 31 |  | I usually buy fair trade products to contribute to a better world. |
